# Lyme Disease in the Era of COVID-19: A Delayed Diagnosis and Risk for Complications

**DOI:** 10.1155/2021/6699536

**Published:** 2021-02-13

**Authors:** Cheryl B. Novak, Verna M. Scheeler, John N. Aucott

**Affiliations:** Lyme Disease Research Center, Division of Rheumatology, Department of Medicine, Johns Hopkins University School of Medicine, Baltimore, MD, USA

## Abstract

We describe a patient with fever and myalgia who did not have COVID-19 but instead had Lyme disease. We propose that the co-occurrence of COVID-19 and Lyme disease during the spring of 2020 resulted in a delayed diagnosis of Lyme disease due to COVID-19 pandemic-related changes in healthcare workflow and diagnostic reasoning. This delayed diagnosis of Lyme disease in the patient we describe resulted in disseminated infection and sixth nerve palsy. We present the use of telemedicine to aid in the diagnosis of Lyme disease and to provide prompt access to diagnosis and care during the ongoing COVID-19 pandemic and in the future.

## 1. Introduction

The worldwide coronavirus-19 (COVID-19) pandemic of 2020 has dominated the attention of healthcare professionals, affecting all arenas of care. Screening for symptoms of COVID-19 (such as respiratory symptoms, fever, myalgia, and fatigue) has been inserted into the front end of all healthcare encounters [1]. A positive symptom screen leads to a prioritized workflow and evaluation which often includes COVID-19 testing [2] and quarantine, pending the test result. During the period of quarantine, evaluation for other causes of the patient's symptoms is often deferred, risking delayed diagnosis and complications [3]. The impact of this dramatic shift in healthcare delivery is only beginning to be understood but includes decreased ambulatory visits with outpatient providers [4]. In addition, the COVID-19 pandemic has led to the dramatic adoption of telemedicine for the delivery of healthcare [5]. It is not known what impact telemedicine will have on the diagnosis of acute infectious diseases such as Lyme disease.

Acute Lyme disease is a seasonal, tick-borne infection with both localized signs of infection of the skin and generalized so-called “flu-like symptoms” of fever, chills, sweats, malaise, myalgia, and arthralgia. These symptoms of Lyme disease are nonspecific and, in the absence of the recognition of the characteristic erythema migrans rash (EM), can lead to misdiagnosis [6, 7]. Barriers to the accurate identification of the diagnostic EM rash include failing to do a complete skin exam and/or misidentification of the varied appearances of the EM rash [8].

Misdiagnosis and delays in diagnosis of Lyme disease can lead to bacterial dissemination and involvement of distant organ systems, most prominently the neurologic, cardiac, and musculoskeletal systems [9]. Neurologic involvement typically manifests after the first few weeks of untreated infection and can result in meningitis, cranial nerve palsy, and radiculoneuritis. Carditis, though rare, can be life-threatening [10].

We present a case of initially misdiagnosed Lyme disease in the era of COVID-19 to highlight new challenges facing healthcare delivery. The objective of this report is to describe the obstacles for the diagnostician during the COVID-19 pandemic and to discuss the potential beneficial application of telemedicine in the diagnosis of Lyme disease.

## 2. Case Presentation

A 67-year-old man was in good health until early July 2020 when he developed an abrupt onset of chills, body aches, fever, headache, and neck ache. He reported no rhinitis, cough, or shortness of breath. He had been working outdoors in rural Pennsylvania the prior week but had not noted a tick bite. The patient was seen in an urgent care center where the diagnosis of COVID-19 was raised, and a COVID-19 RT-PCR test was ordered. He was discharged with the diagnosis of a viral-like illness and told to quarantine until his COVID-19 test results were obtained.

The COVID-19 test was negative, and over the following 6 weeks, his symptoms slowly resolved, but he developed a new skin lesion on his right upper arm. He was seen in person by a dermatologist who diagnosed an insect bite reaction versus Lyme disease. A Lyme disease serology was ordered. The test returned several days later with a positive ELISA, a positive IgM Western blot with 3 of 3 reactive bands, and 4 out of 10 bands reactive on the IgG Western blot. Doxycycline was given orally at a dose of 100 mg twice a day for seven days.

**Figure 1:**
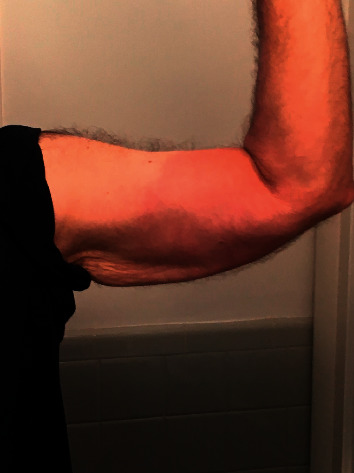
Photograph of patient's erythema migrans skin lesion.

After a week of oral doxycycline, he developed a new symptom of double vision when looking to his right. He continued to complain of headache and neck stiffness with new-onset fatigue. His treatment was switched from doxycycline to oral cefuroxime. An ophthalmology evaluation confirmed a right sixth nerve palsy. The patient was seen in consultation at a Lyme disease telemedicine referral clinic. A detailed history further corroborated a diagnosis of Lyme disease with sixth cranial nerve involvement: an acute febrile illness, occurring during the Lyme disease seasonal peak, and following possible outdoor exposure in an endemic geographic region. Physical examination conducted via telemedicine showed a right sixth nerve palsy. Digital images of his skin lesion that were on his cell phone were reviewed and confirmed the appearance of EM, presenting as an 8 cm oval, red skin lesion, located on the patient's right upper arm (Figure 1). Previously obtained Lyme disease testing confirmed a positive serologic test. Lumbar puncture was obtained to evaluate for meningitis and showed a mildly elevated CSF protein, without pleocytosis. CSF antibodies for Lyme disease were absent. An MRI of the brain was unremarkable. As a result, his treatment was changed from cefuroxime to a higher dose of doxycycline 200 mg twice a day.

A follow-up evaluation after 4 weeks on the higher dose of doxycycline revealed only minimal double vision with extreme right gaze. A review of systems was positive for difficulty initiating sleep, mild anxiety, and mild daytime fatigue. He noted subtle difficulty with his short-term memory. His headache had resolved. Physical exam showed no evidence of residual sixth nerve palsy.

## 3. Discussion

We report a well-documented case of acute Lyme disease with delayed diagnosis and initial confusion with COVID-19. Our case illustrates the overlap of symptoms among disparate infectious diseases and the risk of a narrow approach and focus on COVID-19 in patients with undifferentiated febrile illnesses, such as Lyme disease.

Misdiagnosis of Lyme disease as a viral influenza infection has been previously reported [7]. In the spring of 2020, the COVID-19 pandemic in the Northern Hemisphere coincided with the seasonal onset of tick exposure, presenting a new risk for confusion in the diagnosis of acute tick-borne disease. Symptom screening for COVID-19 is not specific, even if respiratory symptoms are present [11]. Using fever alone as a screening tool is highly nonspecific, and in our case, it led physicians to pursue the diagnosis of COVID-19 to the exclusion of other possible causes. Fever is a common symptom of some noninfectious diseases and most infections. Many of these infections require prompt diagnosis and antibiotic treatment to avoid morbidity and even mortality.

In our reported case, the patient's Lyme disease progressed during the evaluation and quarantine for COVID-19, with the eventual discovery of a skin lesion suspicious for EM. Delays in treatment resulted in dissemination of *B. burgdorferi* infection and subsequent sixth cranial nerve ocular palsy. Cranial nerve six involvement is unusual in Lyme disease compared to the more common seventh nerve palsy [12]. While unusual, the onset of cranial nerve involvement after the initiation of antibiotics can occur. Treatment with oral antibiotics is possible once meningitis has been excluded [13].

This patient's description of his experience re-emphasizes the importance of doctor-patient communication and highlights new issues in miscommunication that may arise during the COVID-19 pandemic. Of primary importance to the patient is the need to investigate symptoms until a specific diagnosis is made. This is especially important in infectious diseases where treatment is often curative and progression of untreated infection can result in significant morbidity or even mortality. This case also illustrates the need to recognize epidemiologic hints for tick-borne disease, such as the patient's presence in an endemic geographic location and the seasonal upswing in Lyme disease vector activity.

The high level of COVID-19 person-to-person infectivity and the resulting pandemic response have led to a prioritized workflow to “rule out” COVID-19 as the first step in healthcare encounters. Delays in testing, especially early in the pandemic, lead to the patient being placed in presumptive quarantine, pending the test outcomes [14]. Once the test returns negative, this may create a risk of premature closure of the diagnostic process. This is further compounded by the lack of ambulatory resources for an in-person evaluation that is so important in the initial diagnosis of infectious diseases. Telemedicine has expanded rapidly to fill this gap, but its potential positive and negative impacts are only beginning to be recognized.

Telemedicine may present specific challenges when the physical exam is central to making an accurate diagnosis. Trends in medicine have drifted away from an emphasis on the physical exam, and the COVID-19 pandemic may contribute to the continued decline in the effective use of the physical exam [15].

At the same time, telemedicine has the potential to improve the patient experience with more convenience and ease of healthcare accessibility. Skin examination and documentation of skin lesions and rashes during tele-visits may provide a crucial hint to the diagnosis of infectious diseases such as Lyme disease and avoid delays in treatment. Live video platforms can be supplemented by cell phone images previously obtained by the patient. Computer-assisted prescreening of skin findings may augment the nonexpert clinician's ability to appropriately triage patients with Lyme and other tick-borne diseases in the future [16].

Limitations of this case report include the inability to confirm our serologic diagnosis with culture or polymerase chain reaction (PCR) tests for *Borrelia burgdorferi*, the etiologic agent of Lyme disease. Culture of *Borrelia burgdorferi* is not available in clinical laboratories, and tissue for PCR is insensitive for the diagnosis of central nervous system Lyme disease and only useful in cases where joint fluid can be obtained.

In conclusion, viral-like symptoms such as fever, myalgia, and fatigue in the absence of respiratory symptoms and in the appropriate epidemiologic setting should prompt suspicion for Lyme disease. Although COVID-19 has overlapping symptoms with other conditions, the need to exclude COVID-19 infection should not distract the clinician from simultaneous evaluation for infectious diseases that will benefit from early diagnosis and prompt treatment. Telemedicine may remain a useful option for the evaluation of acute Lyme disease even after the COVID-19 pandemic subsides.

## 4. Patient Perspective

Written informed consent to publish the case details was obtained from the patient.

“When I first sought treatment for my fever and chills, the clinical team did basic testing to address my health status; this was negative. They did not suggest further testing or give input on what could have been the cause of the chills, muscle aches, etc. The only concern they expressed was the possibility of COVID-19, but since I did not have lung or nasal congestion, they did not see that as highly likely. The symptoms were clearing up at this point as well. In retrospect, I did not ask sufficient questions during this phase to pursue a definite diagnosis.

When weeks later the rash occurred on my arm, I saw a dermatologist who had me tested for Lyme disease. I was not aware of the possible symptoms that could still occur after the antibiotics were started. The double vision and neurological issues were quite alarming. As I sought further medical input (but did not take the suggested step to seek treatment at an emergency room), an out-of-network physician was engaged. This physician did not seem very concerned about the double vision condition I had. This was puzzling, as supposedly, this physician had experience with treating Lyme disease. After my wife repeatedly expressed concerns to his staff, they did start (after several weeks elapsed) to suggest neurological/MRI tests.

My wife sought input on where we could have further medical services and more input in addressing my condition. As the dermatologist had recommended, had we gone to the emergency center, we would have received valuable input from Johns Hopkins. So we sought services from Johns Hopkins Medicine.

As Johns Hopkins undertook review of my situation, treatment started to address Lyme disease symptoms in a detailed manner. The process was undertaken to address the neurological conditions and testing with a lumbar puncture. Antibiotics were changed. Johns Hopkins provided input on how the testing and my health conditions would progress. This was a positive step in the treatment process and assisted my understanding of the situation I was encountering and encouraged me that I would most likely regain my vision.

In my case, once the rash was observed, Lyme disease was considered to be the cause of my illness. However, the rash did not take place until more than 5 weeks after the initial symptoms were encountered. Had Lyme disease been considered in the first stages, perhaps treatments would have prevented the neurological/vision problems that developed later.

I have spoken to several former business colleagues about my experience with Lyme disease. Their comments were similar; friends/family members who had Lyme disease in past years expressed the same complaint: Lyme disease was not tested for in the first stages of the illness because of the lack of a rash. Their stories were remarkably similar to mine. They experienced health problems before Lyme testing was finally considered and done.”

## Data Availability

No data were used to support this case report.
